# Genome-Wide Analysis of Seed Acid Detergent Lignin (ADL) and Hull Content in Rapeseed (*Brassica napus* L.)

**DOI:** 10.1371/journal.pone.0145045

**Published:** 2015-12-16

**Authors:** Jia Wang, Hongju Jian, Lijuan Wei, Cunmin Qu, Xinfu Xu, Kun Lu, Wei Qian, Jiana Li, Maoteng Li, Liezhao Liu

**Affiliations:** 1 College of Agronomy and Biotechnology, Southwest University, Beibei, Chongqing, China; 2 Institute of Resource Biology and Biotechnology, College of Life Science and Technology, Huazhong University of Science and Technology, Wuhan, China; NSW Department of Primary Industries, AUSTRALIA

## Abstract

A stable yellow-seeded variety is the breeding goal for obtaining the ideal rapeseed (*Brassica napus* L.) plant, and the amount of acid detergent lignin (ADL) in the seeds and the hull content (HC) are often used as yellow-seeded rapeseed screening indices. In this study, a genome-wide association analysis of 520 accessions was performed using the Q + K model with a total of 31,839 single-nucleotide polymorphism (SNP) sites. As a result, three significant associations on the *B*. *napus* chromosomes A05, A09, and C05 were detected for seed ADL content. The peak SNPs were within 9.27, 14.22, and 20.86 kb of the key genes *BnaA*.*PAL4*, *BnaA*.*CAD2/BnaA*.*CAD3*, and *BnaC*.*CCR1*, respectively. Further analyses were performed on the major locus of A05, which was also detected in the seed HC examination. A comparison of our genome-wide association study (GWAS) results and previous linkage mappings revealed a common chromosomal region on A09, which indicates that GWAS can be used as a powerful complementary strategy for dissecting complex traits in *B*. *napus*. Genomic selection (GS) utilizing the significant SNP markers based on the GWAS results exhibited increased predictive ability, indicating that the predictive ability of a given model can be substantially improved by using GWAS and GS.

## Introduction

Increasing seed oil content has always been an important goal in the breeding of *B*. *napus*. Research has demonstrated that the yellow-seeded rapeseed is a desirable variety because of its quantities of oil and protein compared with the black-seeded rapeseed [1] as well as the association of the yellow seed color with a thinner seed coat [2], lower fiber content, and reduced amounts of anti-nutrients (lignin) [3]. Moreover, these characteristics make rapeseed meal a valuable source of high-quality feed. Therefore, the selection of a stable yellow-seed trait is one of the most essential breeding objectives for this crop. However, the development of yellow-seeded *B*. *napus* varieties with increased seed oil content and improved canola meal quality (i.e., lower fiber and higher protein contents) have been limited due to insufficient understanding of the genetic mechanisms underlying the formation of seed coat color in this species [4] and to the considerable influence of the environment on the yellow-seed trait [5]. Therefore, the reduced fiber content and thinner seed coat of the yellow-seeded varieties are considered important screening indices for breeding this trait.

Genome-wide association study (GWAS) is rapidly becoming the dominant paradigm for investigating the genetic basis of natural phenotypic variations. Although GWAS have been primarily used for research on human diseases, these studies have also been successful in mapping causal variants in many other organisms [6], including crop plants. The application GWAS to detect quantitative trait loci (QTLs) controlling complex traits has become a popular approach for studying key characteristics of crop plants [7], such as rice [8], maize [9], wheat [10], barley [11], and potato [12]. Association mapping has also been effectively performed in *B*. *napus*. Thus far, due to the complexity of its genomic structure and the lack of high-quality molecular markers, the population structure and linkage disequilibrium (LD) of rapeseed (*B*. *napus* L.) are not well understood compared to those of other crop species [13]. The first association studies based on traits and markers in *B*. *napus* began in 2008. Based on orthologs of candidate genes for glucosinolate biosynthesis in *Arabidopsis*, 51 gene-linked simple sequence repeat (SSR) alleles were found to be associated with seed glucosinolate content in two sets of *B*. *napus* germplasms, with 94 and 46 genotypes separately [14]. Subsequently, numerous *B*. *napus* association analyses have been implemented [15–24]. The findings of these investigations indicate that it is feasible to develop molecular breeding markers related to agronomic traits found in association studies in order to analyze the genetic basis of complex agronomic traits in *B*. *napus*. With the development of the GWAS research method, the genome sequence of *B*. *napus* was completed [25], and the technology was improved to dramatically reduce the cost of the high-throughput single-nucleotide polymorphism (SNP) chip, thus making the GWAS method popular in rapeseed breeding research.

Light seed color and low fiber content are thought to coincide because the biochemical pathways leading to lignin and pigment synthesis have common precursors, such as *p*-cumarate [26]. Moreover, a correlation between seed color and fiber composition has been observed, particularly with the amount of acid detergent lignin (ADL) [27–29]. The lower seed hull content (HC) in yellow-seeded *B*. *napus* is partially attributed to reduced fiber quantity and the reduction of the palisade layer to half to two-thirds of its thickness compared with that of black-seeded varieties [30]. A major quantitative trait locus (QTL) influencing seed ADL content has been mapped to chromosome A9 of *B*. *napus* [27–29,31]. The *Bna*.*CCR1* homolog may interact with neighboring homologs of a *CAD* gene family member known to regulate seed-coat phenylpropanoid biosynthesis in *B*. *napus*. To date, only a few QTL reports, including some from our lab, have focused on seed HC [32–34], which is likely to be controlled by a number of genes and is therefore appropriate for QTL analyses [26]. The biosynthesis of lignin and its phenylpropanoid precursors in xylem and stem tissues has been studied extensively in numerous model and crop plants. In contrast, seed-coat phenylpropanoid biosynthesis is less understood, although seeds from many crops play a huge role in both livestock feeding and human nutrition [28]. In this study, we used a collection of 520 cultivars for association mapping and performed a GWAS for seed ADL content and HC. The aim of this work was to identify SNPs that are significantly associated with the quantity of seed ADL content and HC in mapping populations, to compare the findings with those from previous studies concerning the QTL mapping of seed ADL content [27–29,31], and to identify for candidate genes governing potentially important traits for improved *B*. *napus* breeding.

## Materials and Methods

### Field experiment and traits measurements

A total of 520 *B*. *napus* germplasm resources (98 yellow-seeded, 201 black-seeded, and 221 with intermediate colors) (S1 Table), collected from China spring and semi-winter accessions, were cultivated under natural growing conditions in the experimental farm of the Chongqing Engineering Research Center for Rapeseed, Southwest University in Beibei, Chongqing, China (106.40°E, 29.80°N) for two consecutive years. The accessions were arranged in a randomized complete block design with three replicates. In the growing periods from September 2012 to May 2013 (referred to as 2013) as well as September 2013 to May 2014 (referred to as 2014), each line was planted in two rows of 10 plants per row, with 30 cm between rows and a distance of 20 cm between plants within each row. Open pollinated seeds were collected from five randomly-chosen plants in each line at maturity for seed HC and NIRS analysis.

The determination of seed HC was performed as described by Dimov *et al*. [35]. Seed hulls were separated from the embryos using a dissecting needle and tweezers after the seeds were imbibed in water for 24 h. Both fractions were dried at 105±2°C for 2 h before their dry weights were measured. The seed HC (in %) per sample of 50 seeds (approx. 200 mg) was determined. In addition, 13HC and 14HC represent the seed HC in 2013 and 2014, respectively.

The quantity of ADL in the seeds was estimated using near-infrared reflectance spectroscopy (NIRS) with an NIR System 6500 and the WinISI II software (FOSS GmbH, Rellingen, Germany). Before scanning, the system was warmed up for at least 50 min. Then, the naturally dried samples were transferred into the sample cup. The sample was pressed gently into the sample table of the spectrometer and then scanned. The spectra between 1,100 and 2,498 nm were recorded, registering log (1/R) absorbance values at 2-nm intervals for each sample. The phenotypic values of the ADL content (% seed dry mass) were extrapolated from NIR spectra using NIR calibrations developed in our lab specifically for the measurement of these traits (fiber content) in *B*. *napus*. The standard error of the calibration (SEC), the R-squared value (RSQ), the standard error of cross validation (SECV), the standard error of prediction corrected for bias SEP(c), and the value of 1 minus the variance ratio (1-VR) in this NIR calibration were 0.195, 0.979, 0.227, 0.172, and 0.971, respectively. The NIR-derived estimates for seed ADL content were averaged over three technical replicates.

### Genome-wide association analysis

In these lines, 5,000 SNPs (minor allele frequency [MAF] ≥ 0.2) were utilized to evaluate the population structure, and the 520 rapeseed lines were classified into two groups according to the results of the structure analysis. The relative kinship analysis revealed that the population of *B*. *napus* had a null or weak relationship, with greater than 80.9% pairwise relationship estimates between lines; values less than 0.05 and 55% were considered equal to 0. The level of LD in the rapeseed panel was low, with a distance of LD decays within 0.5 to 1 cM at the genomic level.

A trait—SNP association analysis was performed using the Q + K model with a total of 31,839 SNP sites (miss data < 20%, MAF ≥ 0.05); the Q + K model was implemented via a mixed linear model (MLM) [36,37] by a variance component estimation in TASSEL 5.1 [38]. To avoid the excessive correction from Bonferroni and relatively loose correction from 5% FDR [39], we performed a correction for multiple hypothesis testing by controlling the false discovery rates (FDRs) of 1% in this study. All *p*-values of the association analysis results were ranked from small to large. False discovery rates (FDRs) were calculated as [(m×P)/n]×100% [40], where m is the total number of SNPs, P is the *p*-value threshold for detecting significant association, and n is the total number of significant associations per trait. If *p* < 0.01 after the rectification, then the association between a SNP locus and a target trait was considered significant. We used the R package qqman [41] for Manhattan plots and quantile-quantile plots.

Haplotype blocks were constructed via the four-gamete rule with Haploview 4.2 [42]. The parameters were set as follows: the Hardy-Weinberg *p*-value cutoff was 0.001, the minimum genotype was 75%, the MAF was 0.05, and the maximum number of Mendel errors was 1.

### LD analysis for QTL determination interval

As described by Lund *et al*. [43], the analysis method mainly estimated the significant SNPs. In our populations, the mean linkage disequilibrium (r^2^) between adjacent SNPs was 0.23, and the median was 0.09. LD > 0.1 was used as the determination standard. Each QTL determination interval was recorded as an R-QTL. The LD value between the first left SNP and the significant SNP was computed, with the significant SNP utilized as a reference standard. If LD < 0.1, no further calculations were performed, and this SNP was accepted as the left border of the R-QTL. If LD > 0.1, then the LD value between the next SNP and the significant SNP was calculated. The analysis continued until the LD of a SNP and the significant SNP reached < 0.1, at which point the SNP was accepted as the left border of the R-QTL. The same operation was performed for the right borders of the R-QTLs. When two significant SNPs were adjacent, the SNP on the left side was associated with the left R-QTL, whereas the SNP on the right side was associated with the right R-QTL based on the approach applied to determine the R-QTL interval boundaries of the left and right sides, respectively.

### Genome-wide prediction of seed ADL content and HC

We utilized the R package rrBLUP [44] for genome-wide prediction using ridge regression. To assess the effects of the varying sizes of the training populations, we randomly employed 40 to 80% of the experimental materials. To evaluate the potential of improving the performance of genome-wide prediction by selecting the most informative markers, we compared the predictive abilities of all SNP markers. An equal number of significant SNP markers were chosen randomly, and the significant SNP markers were selected based on their GWAS significance within the training population [45].

### Statistical analysis

Analyses of the means and coefficients of variation and the correlation of the evaluated traits were performed with SPSS 17.0. The effects of genotype, environment, and genotype by environment interaction (G × E) on phenotypic variation were assessed using PROC GLM of SAS 9.2. Upon removal of the outliers, a previously described procedure [46] was implemented to identify the optimal transformation of each trait, thus ensuring that the model assumptions of normally distributed error terms and constant variance were not violated [47]. The normality test of phenotypic traits using the UNIVARIATE procedure of SAS 9.2 was completed, and the Box-Cox transformation of the phenotypic data that did not adhere to a normal distribution was performed with Minitab 16.0.

## Results

### Phenotypic data analysis

Seed ADL content and HC of the *B*. *napus* association panel comprising 520 accessions was measured in three replicates in two consecutive years. As presented in Table 1, extensive phenotypic variations were observed in the descriptive statistics. The seed ADL content, which ranged from 0.44 to 8.07 with an average of 4.37, had a maximum coefficient of variation of 53.10% in 2014. Conversely, the seed HC in 2014, which varied from 9.29 to 20.83 with an average of 14.81, exhibited the lowest coefficient of variation (27.35%). Seed ADL content and HC reached an extremely significant positive correlation in 2013 and 2014; their coefficients were 0.778 and 0.632, respectively (S1 Table). Seed ADL content and HC in 2013 and 2014 manifested a significant positive correlation with correlation coefficients of 0.567 and 0.467, respectively (S2 Table). A two-way analysis of variance (ANOVA) was performed on seed ADL content and HC using the SAS 9.2 software for the GWAS population. The genotype (G), environment (E), and genotype by environment interaction (G × E) exhibited significant effects on all these traits (*p* < 0.01) (Table 1 and S3 Table). The results indicate that seed ADL content was more stable than seed HC in both 2013 and 2014 because their broad-sense heritability coefficients were *h* ^*2*^ = 88.62% and 75.23%, respectively.

**Table 1:** Phenotypic variations in seed ADL content (ADL) and seed hull content (HC) in the *B*. *napus* panel.

Traits	Range	Mean ±SD	CV(%)^a^	G	E	G×E	*h* ^2^ ^b^
13ADL	0.85–7.66	4.42±2.16	48.87	**	**	**	88.62%
14ADL	0.44–8.07	4.37±2.32	53.10				
13HC	9.40–25.50	14.67±4.38	29.86	**	**	**	75.23%
14HC	9.29–20.83	14.81±4.05	27.35				

** The values are significant at P < 0.01 for the effect of genotype (G), environment (E) and genotype by environment interaction (G×E) on phenotypic variance estimated by two-way ANOVA.

^a^ CV is an abbreviation of coefficient of variation, which was estimated as the ratio of the standard deviation to the mean of all accessions.

^b^ *h* ^*2*^ is broad-sense heritability; *h* ^*2*^ = *б* ^2^_g_/(*б* ^2^_g_+*б* ^2^_ge_/*n*+*б* ^2^_e_/*nr*)×100%, where *б* ^2^_g_ is the genetic variance, *б* ^2^_ge_ is the variance due to the G × E interaction, *б* ^2^_e_ represents the residual error, *n* is the number of environments (years), and *r* is number of replicates [48].

Upon removal of the outliers, a four-trait normality test was conducted using the SAS 9.2 UNIVARIATE procedure, and the normal distribution was measured across skewness and kurtosis. The analysis results indicate that 13ADL (skewness: -0.279; kurtosis: -0.024) and 14ADL (skewness: -0.021; kurtosis: -0.247) conformed to the normal distribution, but 13HC and 14HC did not. Thus, we first performed a Box-Cox data transformation of the two phenotypes (2013HC and 14HC) using the Minitab 16.0 software for GWAS to adhere to or approximate a normal distribution in the next step. The software applied the data transformation depending on the lambda value, and the obtained best Box-Cox transformation lambda values of 13HC and 14HC were 0.16 and 0.77, respectively. Through the Box-Cox transformation, 13HC and 14HC reached normal or obviously superior levels than those before the raw data transformation (S1 Fig).

### Genome-wide association studies for seed ADL content and HC

**Fig 1:**
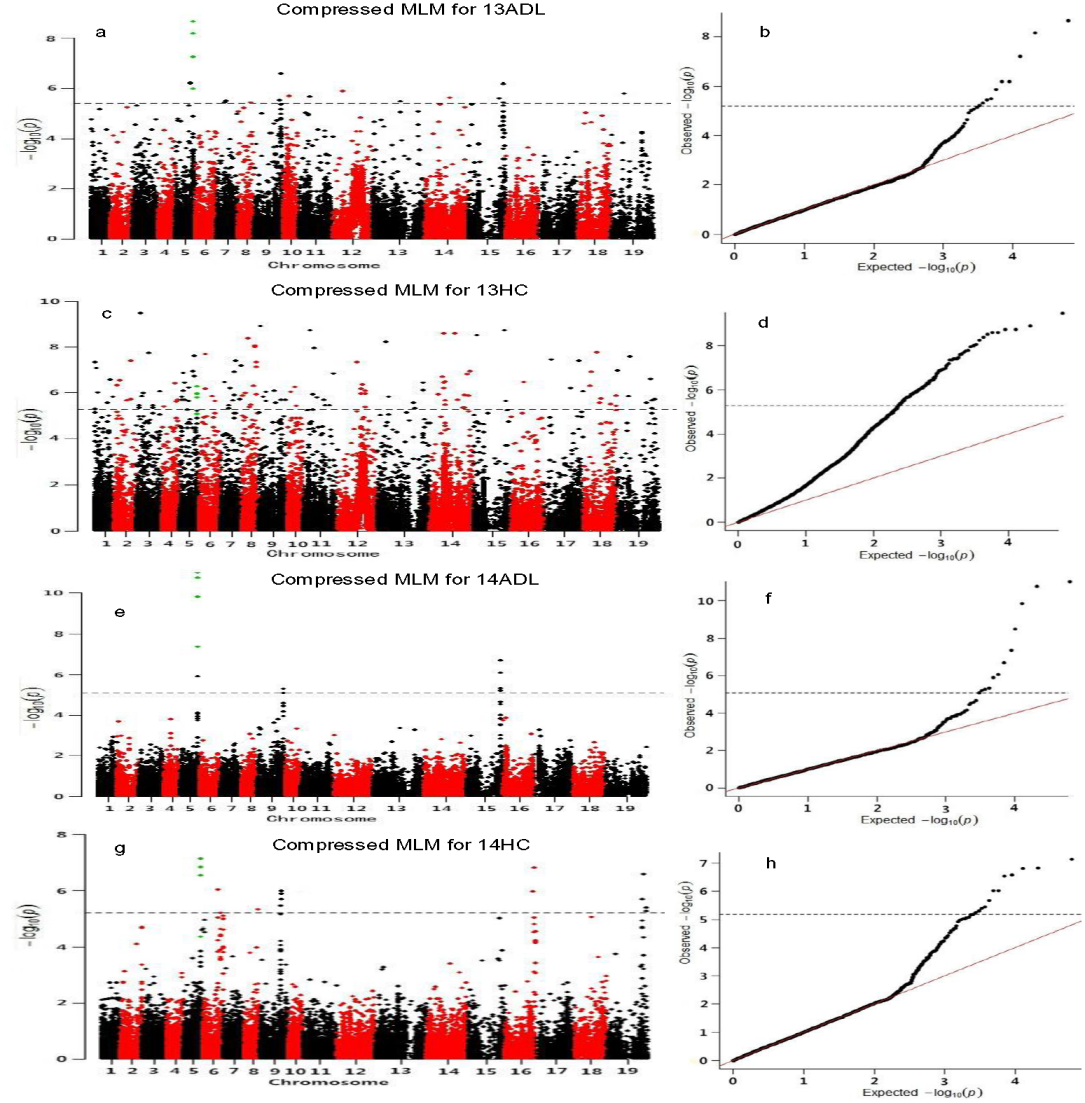
Genome-wide association studies of seed ADL content (ADL) and seed hull content (HC). Manhattan plots of the compressed MLMs for ADL and HC. Negative log_10_-transformed *p*-values from a genome-wide scan are plotted against position on each of the 19 chromosomes. The black horizontal dashed line indicates the genome-wide significance threshold, and the green marker is the simultaneously detected locus. (a): 13ADL, (c): 13HC, (e): 14ADL, (g): 14HC. Quantile-quantile plot of the compressed MLMs for ADL and HC, (b): 13ADL, (d): 13HC, (f): 14ADL. (h): 14HC.

**Fig 2:**
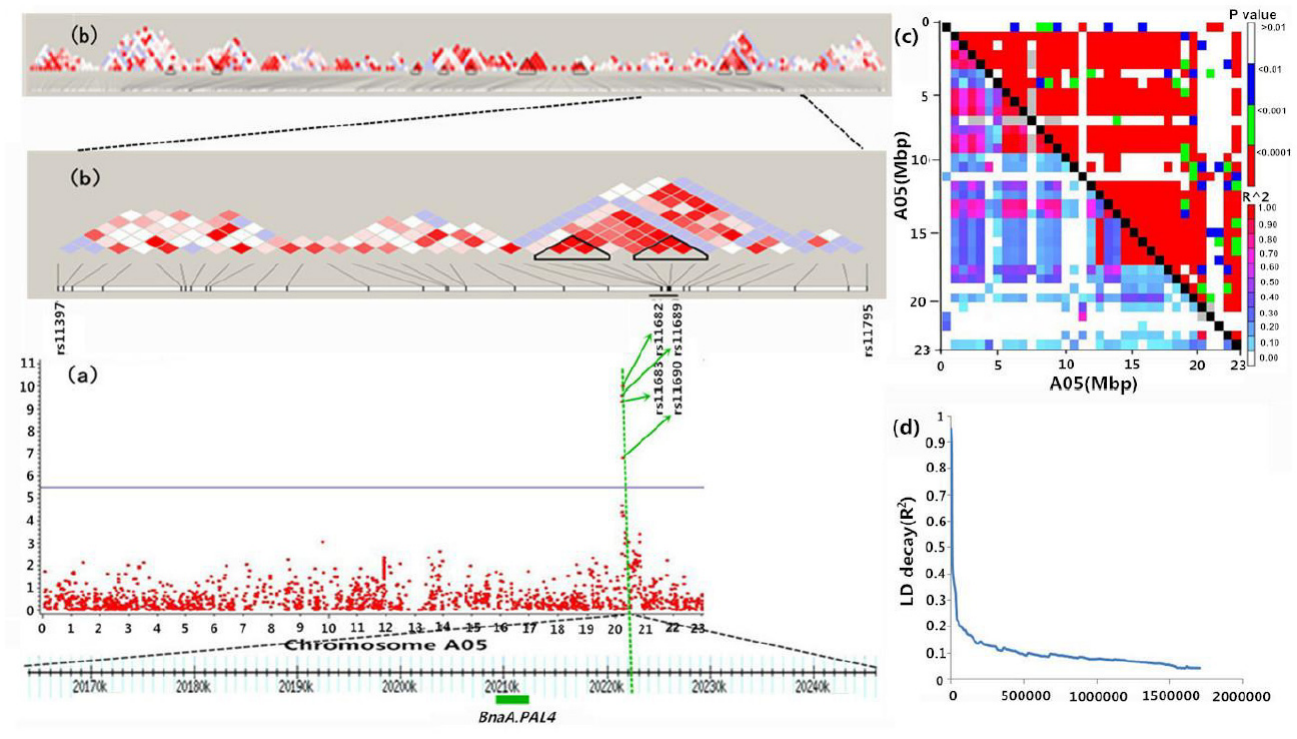
Genome-wide association scan for seed ADL content and the genomic landscape of the major locus on A05. (a) Association signals of ADL (2014) values on A05. The top of the panel shows an R-QTL region based on its significant SNPs, the positions of which are indicated by vertical green lines. Negative log10-transformed *p*-values from the compressed MLM are plotted on the vertical axis. The blue horizontal line indicates the 1% FDR-adjusted significance threshold (6.27 × 10^−6^); the bottom of the panel indicates the related candidate genes marked with green boxes in the R-QTL region. One previously identified gene, *PAL4*, was significantly associated with seed ADL content. (b) The distribution of the linkage disequilibrium (LD) blocks of the major locus on A05. (c) LD analysis of A05. (d) The LD decay of A05.

Using a unified mixed linear model that controlled the population structure and relative kinship, the removal of low-quality SNPs and those with a MAF ≥ 0.05 yielded 31,839 SNP data sets for association analysis. Significant SNP—trait associations at a 1% FDR were found for four traits: 13ADL, 13HC, 14ADL, and 14HC. The Manhattan plots of these traits are presented in Fig 1. The compressed MLM approach, which considered the genome-wide patterns of genetic relatedness, greatly reduced false positives, as illustrated in the quantile-quantile plots (Fig 1b, 1d, 1f and 1h). As detected by the Q + K models, in the ‘pseudomolecules’ of *B*. *napus* (Fig 1a, Table 2 and S4 Table), the seed ADL content (13ADL and 14ADL) was associated with three common significant regions located at 20.2 Mb, 29.8 Mb, and 40.2 Mb in the A05, A09, and C05 chromosomes, respectively. The peak SNP locus on A05 (rs11682; 20,222,542 bp; *p*-values was 2.1×10^−9^ and 1.75×10^−11^ in 2013 and 2014, respectively) was 9.27 kb from the key lignin biosynthesis gene *BraA*.*PAL4* (Fig 2 and Table 3). This peak SNP locus (rs11682) explained 9.4% and 13.9% of the total phenotypic variance for 13ADL and 14ADL, respectively, based on R^2^ values. Regarding seed HC, a total of 150 and 17 association signals were identified during the two experimental years with a 1% FDR from the compressed MLM, respectively (Table 2, S4 and S5 Tables). Three common significant regions for 2013 and 2014 were located at 20.2 Mb, 16.5 Mb, and 33.0 Mb in chromosomes A05, A08, and A09, respectively. The 150 association signals in 2013 were distributed across all chromosomes, and strong association signals with *p* < 5.0×10^−9^ were noted in the physical regions of 4.2 Mb of A03, 2.1 Mb of A09, 7.5 Mb of C01, 35.4 Mb of C05, 14.3 Mb of C04, and 7.3 Mb of A09, accounting for 10.5%, 9.9%, 9.75%, 8.9%, 9.5%, and 9.3% of the phenotype variation, respectively (S5 Table).

**Table 2:** Genome-wide significant association signals of seed ADL content and HC. Only 13ADL, 14ADL and 14HC are listed in this table. The significant association signals of 13HC are presented in S5 Table.

Trait	Chromosome	Position	Major allele	Minor allele	Minor allele frequency	*p*-value	Contribution (%)
13ADL	A05	3207113	G	A	0.45	1.61×10–06	6.369
	A05	16983454	G	A	0.35	6.28×10–07	6.8
	A05	20222542	T	C	0.43	2.21×10–09	9.409
	A05	20222599	C	T	0.44	6.73×10–09	8.892
	A05	20253712	A	G	0.42	5.97×10–08	7.882
	A09	29882802	T	G	0.30	2.90×10–06	6.973
	A09	30668239	A	G	0.41	6.27×10–06	6.666
	A09	31262997	A	G	0.15	2.78×10–06	6.12
	A10	6685826	G	T	0.34	2.02×10–06	6.266
	C01	12655116	G	A	0.19	2.07×10–06	6.254
	C02	12309497	G	A	0.20	1.32×10–06	6.459
	C04	27480077	C	A	0.25	2.44×10–06	5.528
	C05	40211108	C	T	0.31	6.51×10–07	6.111
	C09	15755706	A	C	0.39	1.57×10–06	6.381
14ADL	A05	20222542	T	C	0.43	1.75×10–11	13.92
	A05	20222599	C	T	0.44	1.53×10–10	12.693
	A05	20252156	A	G	0.40	4.37×10–08	9.535
	A05	20253712	A	G	0.42	1.01×10–11	14.232
	A05	20293807	A	C	0.44	1.28×10–06	6.428
	A09	30752816	C	T	0.10	5.18×10–06	5.701
	A09	29882802	T	G	0.30	7.28×10–07	7.386
	C05	39839348	C	T	0.37	4.71×10–06	5.747
	C05	40125110	C	T	0.23	4.70×10–06	6.973
	C05	40132442	T	C	0.23	8.36×10–07	7.914
	C05	40211108	C	T	0.31	1.96×10–07	7.886
	C05	40336201	T	C	0.37	6.33×10–06	6.811
14HC	A05	20222542	T	C	0.43	7.42×10–08	7.768
	A05	20222599	C	T	0.44	1.49×10–07	7.447
	A05	20252156	A	G	0.40	1.48×10–06	8.635
	A05	20253712	A	G	0.42	2.86×10–07	7.148
	A06	17448325	T	C	0.13	9.32×10–07	6.924
	A06	20553941	A	C	0.45	6.52×10–06	6.049
	A08	16530504	A	C	0.09	4.85×10–06	5.854
	A09	24696779	T	C	0.32	3.56×10–06	6.327
	A09	29882802	T	G	0.30	7.28×10–06	6.386
	A09	33496928	T	C	0.29	1.26×10–06	6.531
	A09	33029776	T	G	0.27	9.34×10–07	6.606
	C06	33566777	T	G	0.21	8.64×10–07	6.453
	C06	35525816	C	T	0.06	1.57×10–07	7.421
	C09	41831166	T	C	0.07	2.11×10–06	6.438
	C09	44555029	A	C	0.21	2.63×10–07	7.138
	C09	46608012	G	A	0.08	5.68×10–06	6.172
	C09	46931937	T	A	0.07	4.16×10–06	6.275

**Table 3:** Candidate genes within R-QTL of SNPs most highly associated with seed ADL content.

SNP(s)	Chromosome	Physical interval	Candidate gene(s)
rs11682 rs11683	A05	20169332…20243410	BnA.SEC8 BnaA.PAL4 BnaA05g28490D BnaA.CESA3 BnaA.GPAT5
rs11690 rs11689	A05	20243410…20265627	BnaA05g28570D
rs11282	A05	16961778…17077909	BnaA05g22190D
rs12071	A05	3195182…3222564	BnaA05g06020D BnaA05g06000D
rs11697	A05	20279439…20340965	BnaA.CCR1
rs21364	A09	31213064…31297302	BnaA09g45870D
rs21157	A09	29790101…29917465	BnaA.CAD2 BnaA.CAD3
rs21223	A09	30726019…30755421	CHS1 BnaA09g44870D
rs23199	A10	6630309…6686572	BnaA10g08080D BnaA10g08070D
rs35374	C01	12583623…12804840	BnaC01g18220D BnaC01g18230D
rs29646	C02	12058887…12315563	BnaC02g16830D
rs42895	C04	27304035…27517912	BnaC04g26130D BnaC04g26090D
rs37338 rs37335 rs37333	C05	40099068…40267266	BnaC.CCR1 BnaC05g43300D
rs35157	C05	35235636…35317212	BnaC05g36040D BnaC05g36080D BnaC05g36150D
rs37369	C05	39828480…39936267	BnaC05g42520D BnaC05g42570D BnaC05g42630D
rs37408	C05	40322746…40371177	BnaC05g43530D BnaC.MYB83
rs39358	C09	15753918…15758520	BnaC09g18860D

**Fig 3:**
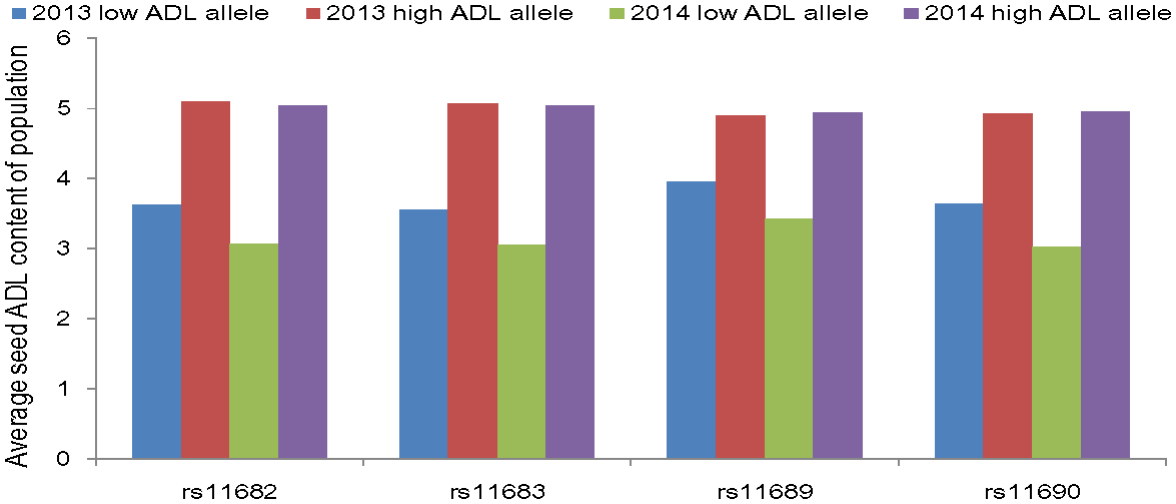
Average seed ADL content of accessions carrying the same allele of the significant SNPs on A05 in *B*. *napus*.

The loci on A05 were repeatedly detected in 2013 and 2014 both for seed ADL content and HC, indicating that a major locus responsible for seed ADL content may be located in this genomic region (Fig 1). LD analysis of A05 was performed using 1,615 SNPs to achieve a comprehensive view of the genomic landscape of this major locus. The LD of A05 over physical distances is depicted in Fig 2. The linkage disequilibrium blocks were unevenly distributed on A05, and the peak SNP associated with seed ADL content was located at one end of A05. The four peak SNPs were located in a similar position on A05 but were not in the same LD block (Fig 2b). Therefore, understanding the LD decay of the A05 genomic region would be facilitated by the determination of candidate genes and beneficial haplotypes. The decay of LD with a physical distance between SNPs occurred at 90 kb on A05 (r^2^ = 0.2) (Fig 2c). The first and third peak SNPs (rs11682 and rs11683) were involved in a 74-kb LD block that encompassed five SNP markers, but the second and fourth peak SNPs (rs11690 and rs11689) were incorporated in a 22-kb LD block that consisted of three SNP markers. This result suggests that the key lignin biosynthesis gene linked with SNPs in A05 might be the major genetic locus responsible for natural variation in rapeseed ADL content. Accessions with a cytosine (C) allele at the first peak SNP (rs11682) manifested, on average, a 40.5% and 64.1% reduced seed ADL content compared with accessions with a thymine (T) allele in 2013 and 2014, respectively. The minor allele (C) was represented in 43% of the 520 accessions. Accessions with a G allele at the second peak SNP (rs11690) had, on average, 45.7% and 62.9% reduced seed ADL content compared with accessions with an adenine (A) allele in 2013 and 2014, respectively. The minor allele (G) was represented in 42% of the 520 accessions. Accessions with a cytosine (C) allele at the third peak SNP (rs11683) had, on average, 42.9% and 64.6% reduced seed ADL content compared with accessions with a thymine (T) allele in 2013 and 2014, respectively. The minor allele (T) was represented in 44% of the 520 accessions. Accessions with an A allele at the fourth peak SNP (rs11689) had, on average, 24.1% and 44.1% reduced seed ADL content compared with accessions with a guanine (G) allele in 2013 and 2014, respectively. The minor allele (G) was represented in 40% of the 520 accessions (Table 2 and Fig 3).

### LD analysis for the QTL determination interval and candidate genes potentially related to seed ADL content

We identified the QTL determination interval using the significant association signals of seed ADL content with known key genes of lignin biosynthesis, and a total of 17 R-QTLs were identified (Table 3). The R-QTLs were 4.60 to 256.68 kb in length with an average length of ~95.73 kb in chromosomal regions near the identified loci with an LD decay distance of 0.5 Mb (r^2^ = 0.1). This average determination interval was much smaller than that delineated by traditional linkage mapping. The major locus on A05 was divided into two R-QTLs. The 74.08-kb QTL determination interval contained two peak SNPs (rs11682 and rs11683), whereas the other R-QTL encompassed two additional peak SNPs (rs11690 and rs11689) spanning a 22.22-kb determination interval, which was identical to the result for the LD blocks. Three additional R-QTLs were noted on A05. Within these R-QTL regions bracketed by significant SNPs, a total of 32 potential candidate genes related to the trait were noted (Table 3). The peak SNP contained two identical genotype SNPs (rs11682 and rs11683) on A05 located in gene *BnaA05g28490D*, the product of which is involved in microtubule-based movement and aligns most closely with *AT3G10310* from *Arabidopsis thaliana*. The key lignin biosynthesis gene *BnaA*.*PAL4* was 9.27 kb from the peak SNP rs11682. In addition, various candidate genes responsible for lignin biosynthesis or seed-coat development were identified in the R-QTL of the peak SNP rs11682. *BnaA*.*CCR1* was identified in another R-QTL associated with the significant SNP rs11697. Moreover, the peak SNPs rs37333, rs37335, and rs37338 on C05 were located within 168.20 kb of an R-QTL, and *BnC*.*CCR1* was 20.86 kb from rs37338 (Table 3 and S2a Fig).

### The major locus on A09 for seed ADL content detected via GWA mapping compared with that determined by QTL mapping

**Fig 4:**
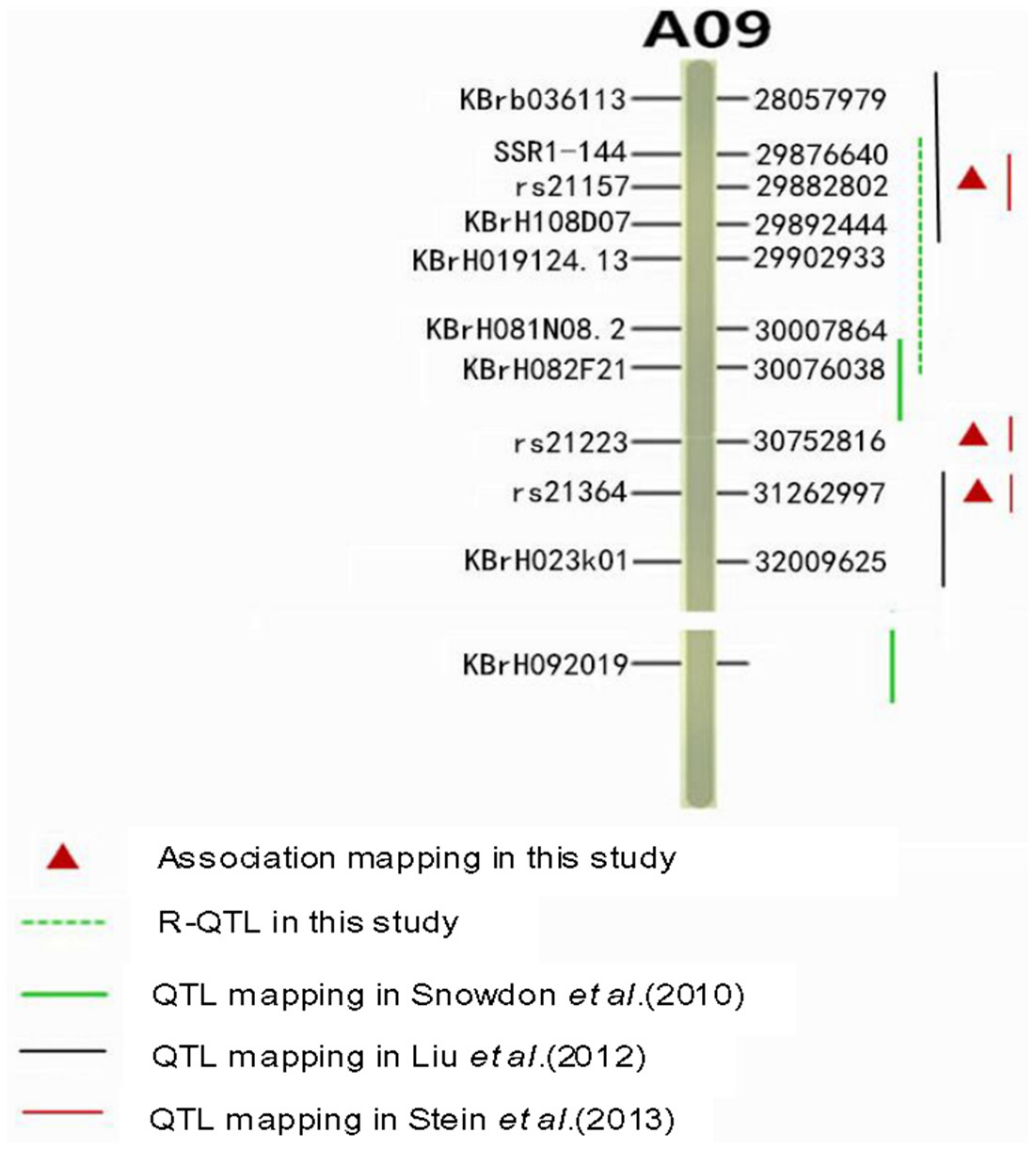
Overlapping or linkage relationships among seed ADL content quantitative trait loci (QTLs) in this and previous studies.

To compare the positions determined by GWA mapping and QTL mapping, we used common markers from previous studies to anchor the latest *B*. *napus* genome (v4.1) via local BLAST searching. The results are presented in Fig 4. In total, five QTLs from QTL mapping and three signals from GWA mapping associated with seed ADL content distributed on chromosome A09 were analyzed. The peak SNP rs21157 was 14.22 kb from *BnaA*.*CAD2/BnaA*.*CAD3* in the marker intervals of ssr1-144 and KBrH108D07, where they overlapped with the QTL that was reported by Snowdon *et al*. [27] and Stein *et al*. [31] based on seed ADL content (S2b Fig). However, the marker KBrH092019, which encompasses the key lignin biosynthesis gene *BnaA*.*CCR1* and was communicated by Liu *et al*. [28,29] and Stein *et al*. [31] was anchored on a scaffold on A09.

In addition, our R-QTLs overlapped with previous QTLs on A09. The R-QTL that contained *BnaA*.*CAD2/BnaA*.*CAD3* located in the determination interval of the peak SNP rs21157 overlapped with previous QTLs reported by Snowdon *et al*. [27] and Stein *et al*. [31] and was only 90.34 kb from the QTL described by Liu *et al*. [28,29]. These results indicate that our methods using GWA mapping generate clear association signal intervals that potentially enable the screening of candidate genes.

### Loci associated with seed HC

**Fig 5:**
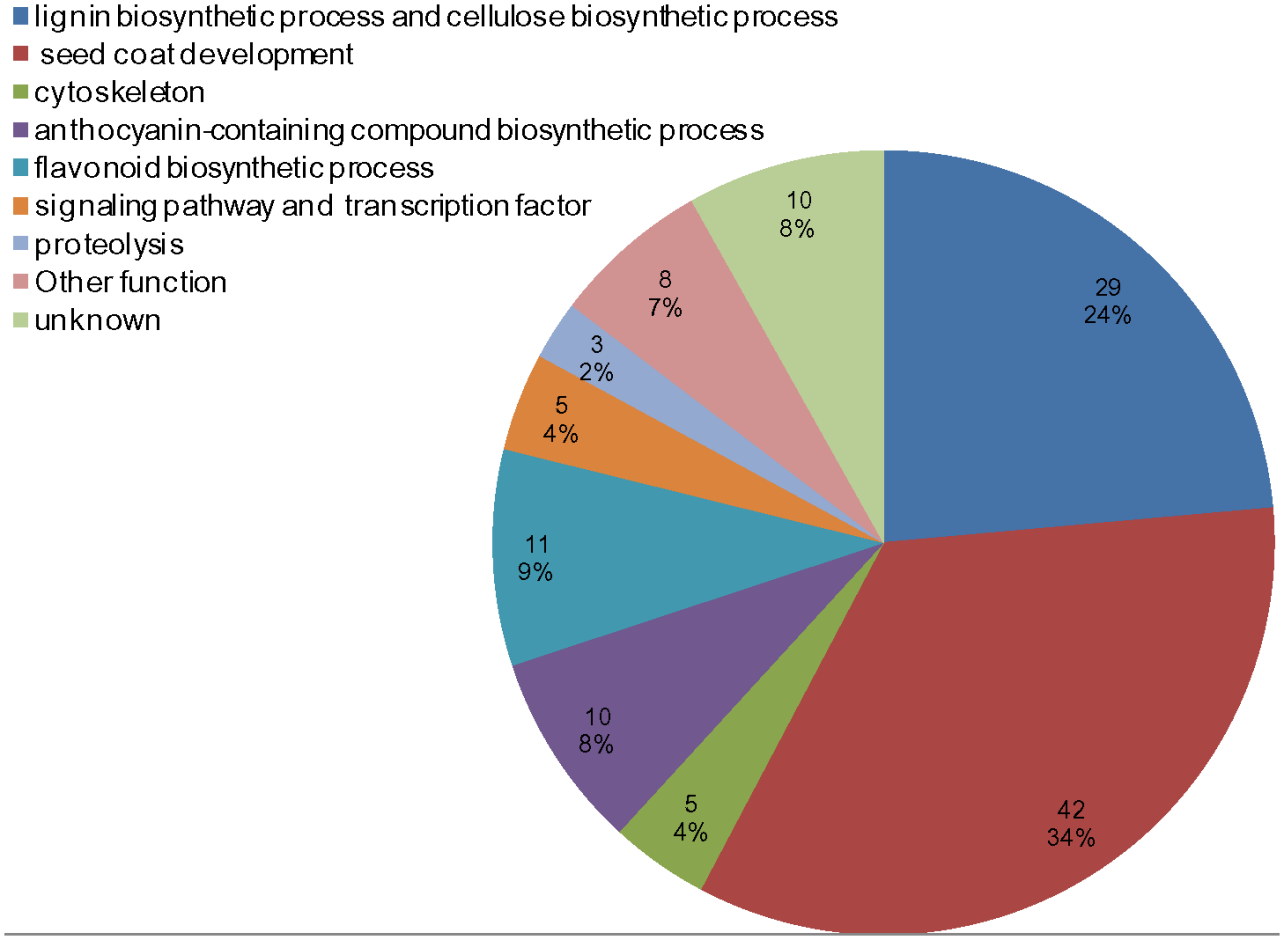
Functional category annotations for 123 candidate genes and their respective percentages identified via GWAS as significantly associated with seed hull content (HC) in *B*. *napus*.

Although 167 loci were highly significantly associated with seed HC in 2013 and 2014, only 4 loci were detected in both years. Given that the genotype and the environment together affect seed HC, this result makes biological sense because the latter had a relatively small value of broad-sense heritability (*h* ^*2*^ = 75.23%). To evaluate whether GWA mapping performed high-efficiency detection, we filtered candidate genes for each identified locus. All potential candidate genes 250 kb upstream or downstream of the lead SNPs (r^2^ = 0.1) of the loci are listed in S6 Table. Among these 123 filtered candidate genes, 68% of these genes associated with seed-coat development and fiber compound biosynthesis, and 17% of these genes were related to the phenylpropanoid pathway of flavonoid biosynthetic processes and anthocyanin accumulation (Fig 5), indicating that our approach can detect candidate genes related to seed HC with a relatively high detection efficiency.

### Genomic selection for seed ADL content and HC

**Fig 6:**
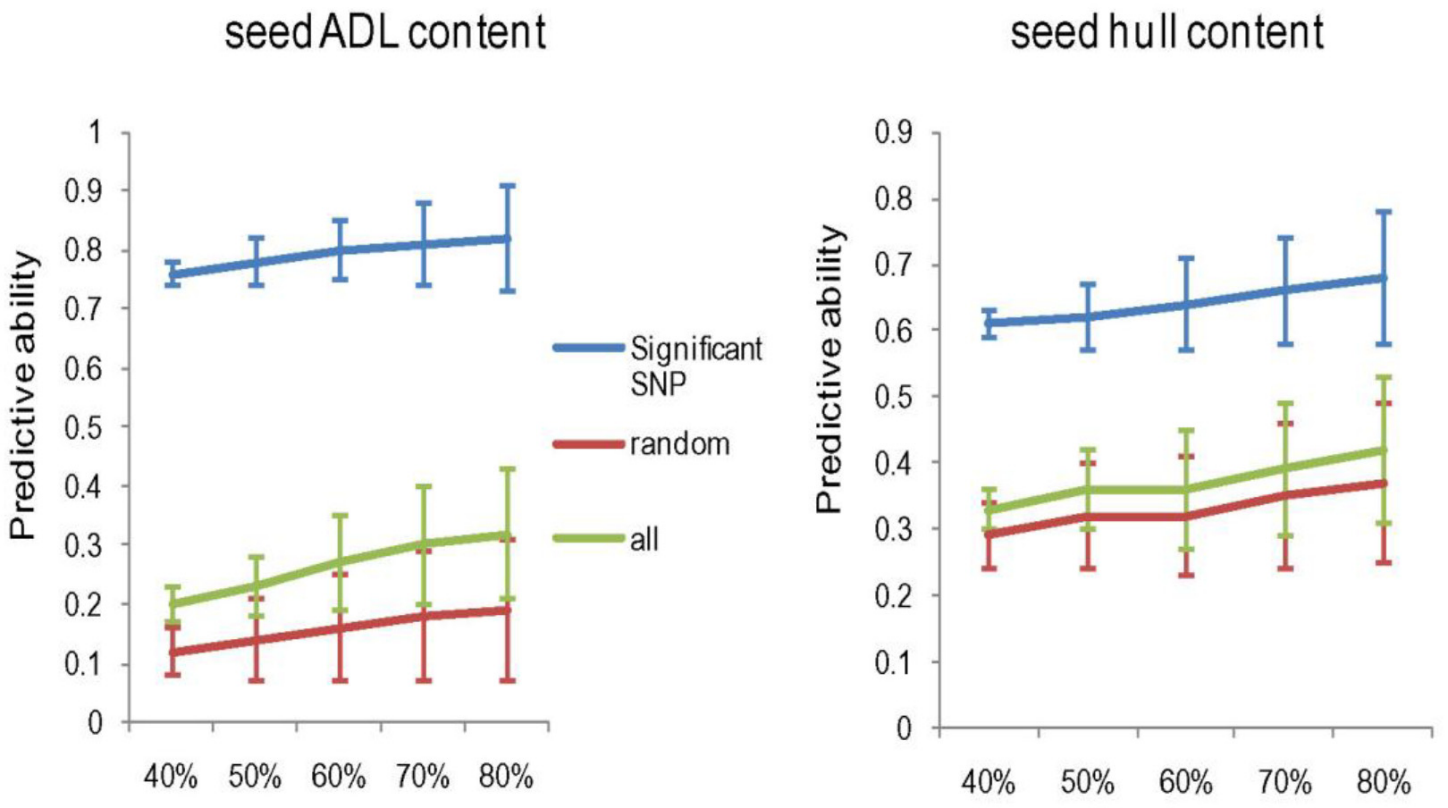
Predictive ability of genome selection with different reference populations (x-axis) and SNP markers (y-axis).

In our study, genomic selection was performed using phenotypic and SNP genotype data. As depicted in Fig 6, we established the choice model with a random 40 to 80% fraction of the experimental materials and all of the 31,839 SNP markers. The predictive ability and standard deviation increased with the size of the reference population. A higher predictive ability and a lower standard deviation were optimal; therefore, 60% of the reference population was selected based on both predictive abilities and standard deviation. All markers were used for the prediction, and the predictive abilities were only 0.27 and 0.36 in seed ADL content and HC, respectively, indicating that not all markers were suitable for efficient prediction. Then, we selected significant association markers identified by GWAS whose predictive abilities were 0.8 for seed ADL content and 0.64 for seed HC, and an equal number of the significant association markers were randomly selected and recorded. The predictive efficiency of the randomly selected associate markers was low, and they manifested a reduced ability to predict seed ADL content and HC. The findings of this examination indicate that significant association markers have a higher predictive ability for seed ADL content and HC. In conclusion, the significant loci detected in GWAS improved the predictive ability for seed ADL content and HC.

## Discussion

Rapeseed seed-coat thickness varies depending on variety and seed-coat color. An anatomical analysis of the seed coat indicates that enlarged cells of the palisade and sponge tissues cause the seed coat of black-seeded rapeseed to thicken, and the color depth of the seed coat is mainly determined by the pigment type and quantity in palisade tissue cells [30]. Reducing seed-coat thickness may decrease or even eliminate the accumulation of pigments contributing to increased oil content output and good production quality. Lignin in yellow-seeded rapeseed is one of the key factors that leads to a seed-coat ratio lower than that of the black-seeded rapeseed [49]. Therefore, reducing seed ADL content can indirectly thin seed coats, not only increasing oil content but also improving the nutritive value of rapeseed meal [29].

The reduced fiber content and thinner seed coat of rapeseed are considered important screening indices for breeding. Previous studies on seed ADL content and HC have been conducted mainly using QTL mapping. Thus, we utilized GWAS for the purposes of this study. The results obtained in the two consecutive experimental years indicated that significant association signals of seed ADL content were found not only on the recognized chromosome A09 but also on chromosomes A05 and C05. We were particularly interested in the significant association signals of ADL on chromosome A05, which were repeatedly noted for seed HC, reflecting the significant positive correlation between seed ADL content and HC. These results provide a theoretical basis for research on the genes affecting seed ADL content and HC. However, many shortcomings should be noted in our study, such as the small population size and low genetic diversity of these landraces within China, both of which can limit the potential of GWAS. The peak signals of the GWAS loci are often close to but not within the range of known genes due to the low density of SNPs around the candidate genes. This work is a preliminary GWAS for seed ADL content and HC in Chinese rapeseed. On one hand, expanding the population size using worldwide accessions is necessary, particularly concerning the yellow-seeded lines. On the other hand, the candidate genes need to be functionally verified via sequence and expression analyses or even gene transformation to confirm whether they are genuine.

The QTLs from preliminary mapping results often spanned ∼10 to 30cM [50] and contained hundreds of genes. Association mapping based on LD has been successfully used for exploring trait-associated loci in animals and plants, but this method cannot provide the confidence interval of statistical significance ensured by traditional QTL mapping. Therefore, association mapping and traditional QTL mapping are complementary methods [18], and their integration will substantially promote the analysis of complex quantitative traits. At present, nested association mapping (NAM) [51] and multiparent advanced generation intercross (MAGIC) [52], based on linkage mapping and GWA mapping are popular methods. If LD exists, it can be detected among the significant SNP markers and QTLs. Thus, we applied the LD analysis method in this study to infer the approximate range of the QTL position through an assessment of the LD degree between the significant SNP markers and their surrounding SNP markers. Our results indicate that the average range of R-QTL was approximately 92.32 kb, which is considerably smaller than that of traditional linkage mapping, and the R-QTLs can therefore provide a valuable reference for the next step of marker-assisted selection. We further analyzed the significant association signals on chromosomes A05, A09, and C05. Our findings indicate that all three R-QTL regions of the three peak SNPs contained genes related to lignin synthesis, particularly the R-QTL containing rs11682 and rs11683. Four key genes, *BnA*.*SEC8*, *BnaA*.*PAL4*, *BnaA*.*CESA3*, and *BnaA*.*GPAT5*, associated with the phenylpropanoid-lignin pathways and seed-coat development were identified in this R-QTL region. Moreover, a comparison of our GWAS and QTL mapping results revealed a common chromosomal region on A09, and a key lignin biosynthesis gene *BnaA*.*CAD2/BnaA*.*CAD3* was discovered in this region. We are particularly interested in the significant association signals on chromosome A05 because it might be a potential locus with enormous importance for seed ADL content. In addition, we previously established that the ADL-related QTLs from QTL mapping were inherited from the yellow-seeded parent. The different seed ADL content signals from GWAS mapping were from diverse seed ADL content accessions. The accuracy of trait scoring is exceedingly important, and the outcomes will directly affect the reliability of GWAS. The identification of major loci in this study will provide the genetic resources and markers needed for the selection of yellow- and black-seeded lines with reduced lignin content and a thinner seed coat.

In this study, we focused on the significant association signals of seed ADL content rather than seed HC given the poor reproducibility of seed HC in the two-year experimental period. All the results reasonably support the finding that the loci detected in our study are located close to candidate genes for controlling seed ADL content and that these tightly associated SNPs are of significant benefit for the design of molecular markers for breeding *B*. *napus* to enhance production and improve its genetics. We propose two explanations for the poor reproducibility of seed HC between the two years in our study. First, the seed embryo was not completely stripped, causing experimental errors. Second, the experimental plants experienced serious crop lodging in 2013, and the seed HC could have been strongly influenced by the decreased seed quality. Furthermore, most significant association signals for seed HC in 2013 were from environmentally specific loci.

Genomic selection (GS) is an important aspect of plant breeding. In this study, we used the R package rrBLUP to conduct a genome-wide prediction for seed ADL content and HC. The results indicate that genomic selection using the significant SNP markers identified via GWAS had a higher predictive ability, indicating that the predictive ability of a given model can be improved by combining GWAS and GS. Molecular marker-assisted selection is the main aim of molecular breeding, using close linked markers for molecular marker-assisted selection can ensure the accuracy of the selection and improve the efficiency of the selection [53]. SSR markers can be developed according to the sequence around the significant associated SNP loci, and can be easily used in laboratory for the low seed ADL content and HC with high seed protein content in *B*. *napus* breeding. Furthermore, the low seed ADL content and HC of rapeseed can be considered the important screening indices for high seed protein content of the defatted meal rather than seed-coat color.

At present, GWAS investigation have entered a post-GWAS world [54]; the most contentious and difficult issues in our GWAS were the interaction analyses of G × G and G × E as well as the analysis of regulatory networks. Further work to analyze the population differentiation index, genetic diversity, and selective sweep of this GWAS population is ongoing, and an analysis for candidate genes can be conducted in the near future. Along with the emergence of new sequencing technologies and the continuing decline in sequencing costs, genome-wide association research will find a wider application in the investigations of *B*. *napus* and other crops.

## Conclusions

With the GWAS Q + K model, significant association signals for seed ADL content were found not only on the recognized chromosome, A09, but also on chromosomes A05 and C05 in two experimental years. A comparison of our GWAS results and those of previous linkage mappings revealed a common chromosomal region on A09 and a major locus on A05 associated with seed ADL content, as detected by GWA mapping. Various key lignin biosynthesis genes were identified in the R-QTLs of seed ADL content based on the LD analysis. In total, 123 candidate genes associated with seed HC were identified by GWA mapping. Greater than 65% of these genes are involved in the phenylpropanoid pathway and are transparent testa genes. The beneficial allele and candidate genes will be useful in rapeseed breeding for achieving the ideal yellow-seeded rapeseed variety through a molecular design approach.

## Supporting Information

S1 Fig The optimal Box-Cox transformation of 13HC and 14HC. (TIF)

S2 Fig Genome-wide association scan for seed ADL content on A05. The top of the panel shows an R-QTL region based on a significant SNP, whose position is indicated by a vertical green line. The negative log10-transformed *p*-values from the compressed MLM are plotted on the vertical axis. The blue horizontal lines indicate the 1% FRD-adjusted significance threshold (6.27 × 10^−6^), and the bottom of the panel shows the related candidate genes with green boxes in the R-QTL region. Two previously identified genes, (a) *CCR1* and (b) *CAD2/CAD3*, were significantly associated with seed ADL content.
(TIF)

S1 Table *Brassica napus* accession information in this study. (XLSX)

S2 Table Correlation analysis for seed ADL content and HC over two years. (DOCX)

S3 Table ANOVA of seed ADL content and HC phenotypes. (DOCX)

S4 Table The sequence data of significant association SNPs for seed ADL content and HC. (XLSX)

S5 Table Genome-wide significant association signals for seed hull content (13HC). (DOCX)

S6 Table Candidate genes near the genomic regions most highly associated with seed HC in *B*. *napus*. (DOCX)
