# Supplementary figures and images for: Genome-Wide Analysis of Seed Acid Detergent Lignin (ADL) and Hull Content in Rapeseed (Brassica napus L.)
Source: PLoS One. 2015 Dec 16;10(12):e0145045. doi: 10.1371/journal.pone.0145045 (PMC4684223; doi:10.1371/journal.pone.0145045)

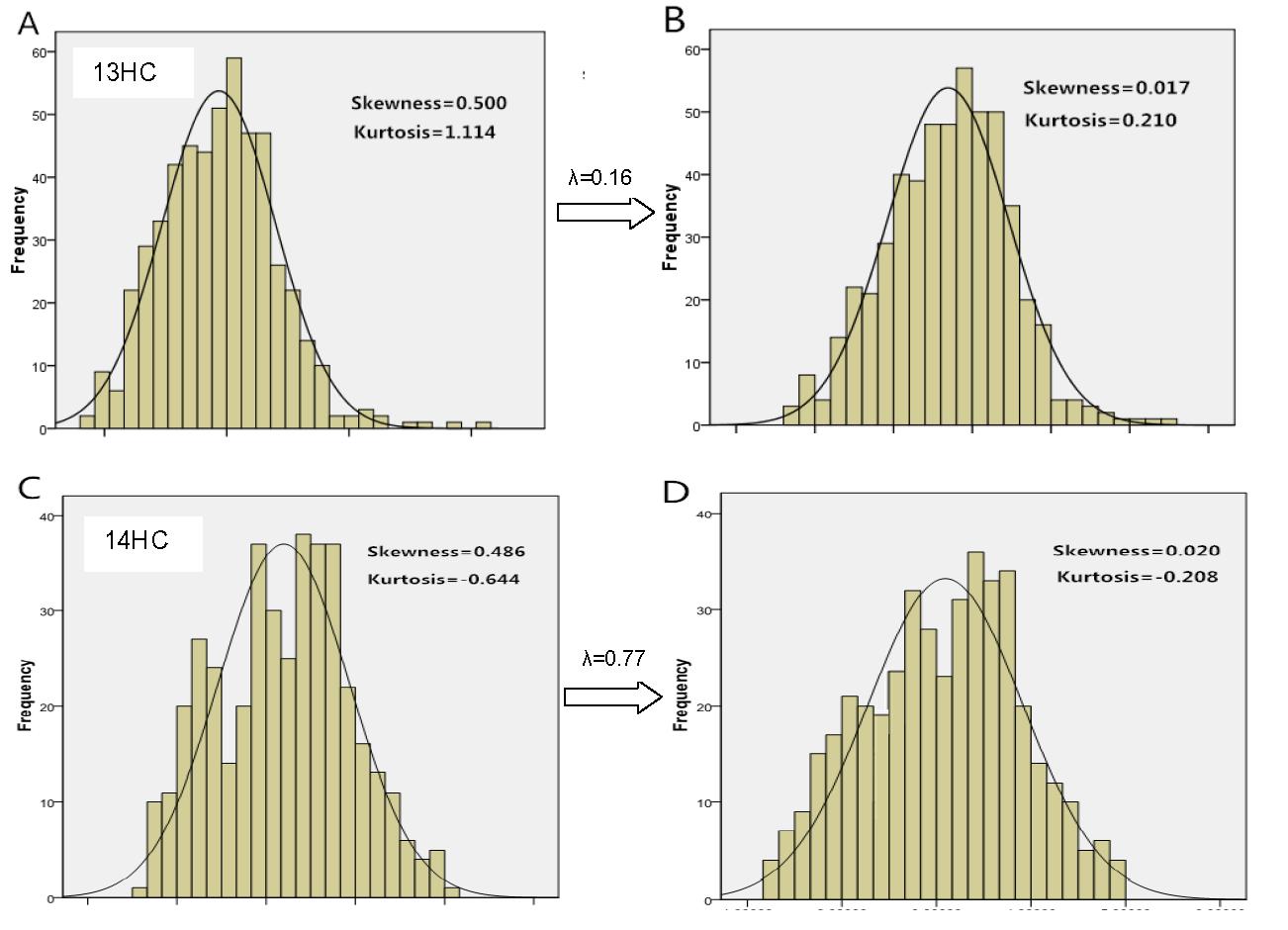

Supplement: S1 Fig — (TIF) [file pone.0145045.s001.tif]

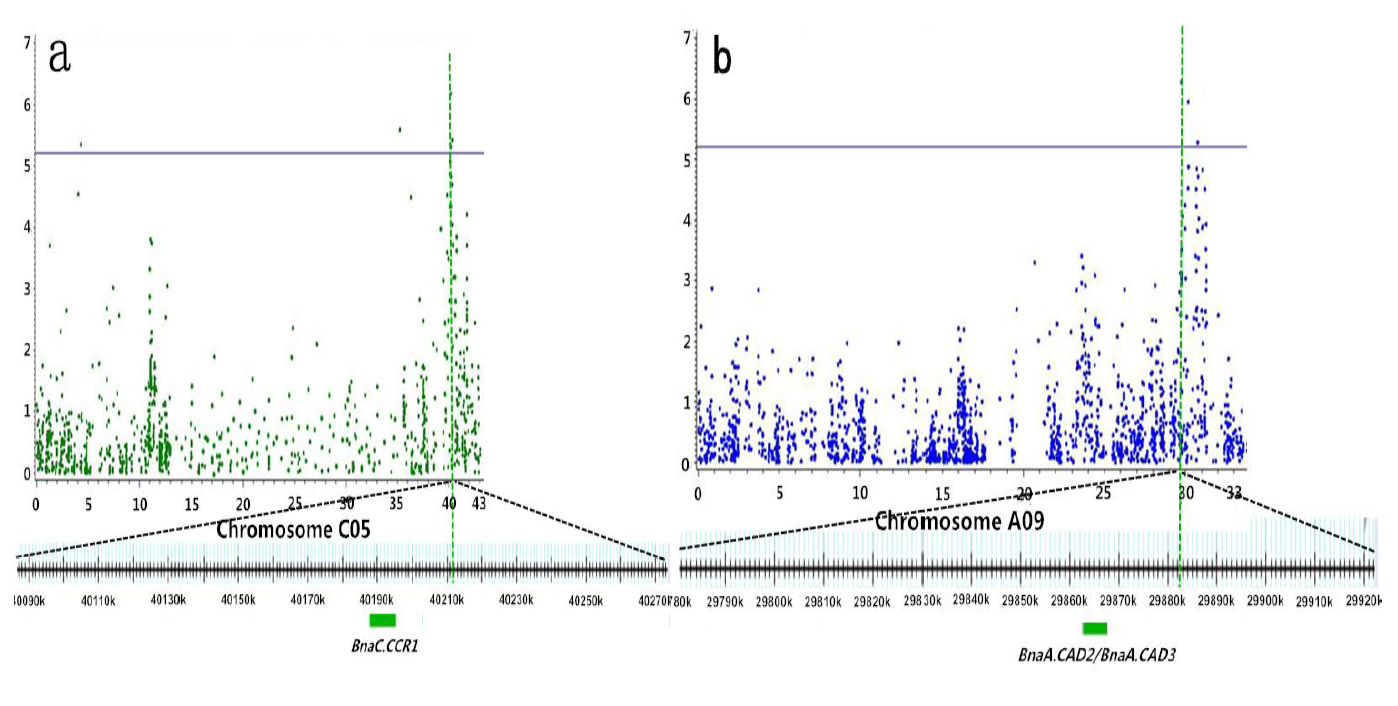

Supplement: S2 Fig — The top of the panel shows an R-QTL region based on a significant SNP, whose position is indicated by a vertical green line. The negative log10-transformed p-values from the compressed MLM are plotted on the vertical axis. The blue horizontal lines indicate the 1% FRD-adjusted significance threshold (6.27 × 10^−6^), and the bottom of the panel shows the related candidate genes with green boxes in the R-QTL region. Two previously identified genes, (a) CCR1 and (b) CAD2/CAD3, were significantly associated with seed ADL content. (TIF) [file pone.0145045.s002.tif]
