# Supplementary material for: Genome-Wide Analysis of Seed Acid Detergent Lignin (ADL) and Hull Content in Rapeseed (Brassica napus L.)
Source: PLoS One. 2015 Dec 16;10(12):e0145045. doi: 10.1371/journal.pone.0145045 (PMC4684223; doi:10.1371/journal.pone.0145045)
Supplement: S2 Table — (DOCX) [file pone.0145045.s004.docx]

**S2 Table Correlation analysis for seed ADL content and HC over two years**

|  | 2013ADL | 2013HC | 2014ADL |
| --- | --- | --- | --- |
| 2013HC | 0.567** |  |  |
| 2014ADL | 0.778** | 0.382** |  |
| 2014HC | 0.546** | 0.632** | 0.467** |

**. Correlation is significant at the 0.01 level (2-tailed).
