# Supplementary material for: Genome-Wide Analysis of Seed Acid Detergent Lignin (ADL) and Hull Content in Rapeseed (Brassica napus L.)
Source: PLoS One. 2015 Dec 16;10(12):e0145045. doi: 10.1371/journal.pone.0145045 (PMC4684223; doi:10.1371/journal.pone.0145045)
Supplement: S3 Table — (DOCX) [file pone.0145045.s005.docx]

**S3 Table ANOVA of seed ADL content and HC phenotypes**

| Trait | source | Df | SS | MS | F value | P value |
| --- | --- | --- | --- | --- | --- | --- |
| ADL | Genotype | 519 | 5488.599 | 10.535 | 311.801 | <0.001 |
|  | Environment | 1 | 15.831 | 15.831 | 468.567 | <0.001 |
|  | Genotype×Environment | 519 | 624.838 | 1.199 | 35.496 | <0.001 |
|  | Error | 2080 | 70.547 | 0.034 |  |  |
| HC | Genotype | 519 | 12403.759 | 23.899 | 220.728 | <0.001 |
|  | Environment | 1 | 14.106 | 14.106 | 130.279 | <0.001 |
|  | Genotype×Environment | 519 | 3072.655 | 5.920 | 54.679 | <0.001 |
|  | Error | 2080 | 225.212 | 0.108 |  |  |
