# Supplementary material for: Genome-Wide Analysis of Seed Acid Detergent Lignin (ADL) and Hull Content in Rapeseed (Brassica napus L.)
Source: PLoS One. 2015 Dec 16;10(12):e0145045. doi: 10.1371/journal.pone.0145045 (PMC4684223; doi:10.1371/journal.pone.0145045)
Supplement: S5 Table — (DOCX) [file pone.0145045.s007.docx]

**S5 Table Genome-wide significant association signals for seed hull content (13HC)**

| Trait | Chromosome | Position | Major allele | Minor allele | Minor allele frequency | P value | contribution(%) |
| --- | --- | --- | --- | --- | --- | --- | --- |
| 13HC | A01 | 1290703 | G | A | 0.32 | 4.57×10^-08^ | 8.156 |
|  | A01 | 1779847 | G | A | 0.33 | 8.61×10^-08^ | 7.859 |
|  | A01 | 17717315 | A | G | 0.32 | 2.75×10^-07^ | 7.314 |
|  | A02 | 19237258 | C | T | 0.27 | 4.08×10^-08^ | 8.209 |
|  | A02 | 5191828 | G | T | 0.35 | 2.86×10^-07^ | 7.295 |
|  | A03 | 4295003 | C | T | 0.30 | 3.44×10^-10^ | 10.475 |
|  | A03 | 14735140 | C | T | 0.27 | 1.77×10^-08^ | 8.603 |
|  | A05 | 17522685 | A | G | 0.37 | 2.46×10^-08^ | 8.448 |
|  | A05 | 15861727 | A | G | 0.29 | 1.16×10^-07^ | 7.72 |
|  | A05 | 4373236 | T | C | 0.29 | 1.41×10^-07^ | 7.627 |
|  | A05 | 16983454 | G | A | 0.35 | 1.86×10^-07^ | 7.497 |
|  | A06 | 7331461 | A | C | 0.38 | 1.99×10^-08^ | 8.549 |
|  | A07 | 16830449 | G | A | 0.27 | 4.00×10^-08^ | 8.219 |
|  | A07 | 23418663 | T | G | 0.30 | 6.73×10^-08^ | 7.974 |
|  | A07 | 15988008 | T | G | 0.26 | 1.25×10^-07^ | 7.684 |
|  | A08 | 7397046 | A | C | 0.31 | 4.13×10^-09^ | 9.291 |
|  | A08 | 14679373 | A | C | 0.26 | 8.62×10^-09^ | 8.943 |
|  | A08 | 14922078 | T | C | 0.27 | 1.00×10^-08^ | 8.871 |
|  | A08 | 17007108 | T | C | 0.24 | 4.64×10^-08^ | 8.149 |
|  | A08 | 17026237 | A | C | 0.27 | 7.50×10^-08^ | 7.924 |
|  | A09 | 2180009 | C | T | 0.27 | 1.24×10^-09^ | 9.862 |
|  | C01 | 7529799 | G | A | 0.24 | 1.82×10^-09^ | 9.679 |
|  | C01 | 12655116 | G | A | 0.19 | 1.13×10^-08^ | 8.814 |
|  | C01 | 34701303 | C | G | 0.16 | 1.43×10^-07^ | 6.911 |
|  | C02 | 22906371 | G | A | 0.23 | 4.42×10^-08^ | 7.446 |
|  | C03 | 9403624 | G | T | 0.27 | 5.89×10^-09^ | 9.123 |
|  | C04 | 14356066 | T | G | 0.31 | 2.51×10^-09^ | 9.528 |
|  | C04 | 27480077 | C | A | 0.25 | 2.51×10^-09^ | 8.761 |
|  | C04 | 44583867 | G | A | 0.27 | 1.14×10^-07^ | 7.016 |
|  | C04 | 42446998 | T | G | 0.29 | 1.56×10^-07^ | 7.579 |
|  | C04 | 14154413 | G | A | 0.31 | 1.94×10^-07^ | 7.478 |
|  | C05 | 35242768 | C | A | 0.25 | 1.89×10^-09^ | 8.892 |
|  | C05 | 4042723 | A | G | 0.28 | 2.97×10^-09^ | 9.447 |
|  | C07 | 8399188 | A | C | 0.32 | 3.56×10^-08^ | 8.274 |
|  | C07 | 40918425 | T | G | 0.38 | 4.13×10^-08^ | 8.203 |
|  | C08 | 16438368 | A | C | 0.30 | 1.70×10^-08^ | 8.623 |
|  | C08 | 9447598 | C | T | 0.24 | 1.23×10^-07^ | 6.979 |
|  | C09 | 15755706 | A | C | 0.39 | 2.56×10^-08^ | 8.429 |
|  | C09 | 994523 | G | T | 0.20 | 1.09×10^-07^ | 7.034 |
|  | C09 | 39518746 | T | C | 0.27 | 2.46×10^-07^ | 7.366 |
