# Supplementary material for: Genome-Wide Analysis of Seed Acid Detergent Lignin (ADL) and Hull Content in Rapeseed (Brassica napus L.)
Source: PLoS One. 2015 Dec 16;10(12):e0145045. doi: 10.1371/journal.pone.0145045 (PMC4684223; doi:10.1371/journal.pone.0145045)
Supplement: S6 Table — (DOCX) [file pone.0145045.s008.docx]

**S6 Table Candidate genes near the genomic regions most highly associated with seed HC in *B. napus***

| SNP | Chromosomes | candidate gene | Gene name | Arabidopsis homologue | Distance(kb) | E-value | Direction |
| --- | --- | --- | --- | --- | --- | --- | --- |
| rs702 | A01 | BnaA01g02580D | COW1 | AT4G34580 | whithin | e-120 | developmental growth |
| rs1295 | A01 | BnaA01g03840D | SQD1 | AT4G33030 | whithin | 0.E+00 | glycolipid biosynthetic process |
| rs2061 | A01 | BnaA01g09330D |  | AT4G18490 | whithin | 7.E-54 | unknown protein |
| rs18 | A01 | BnaA01g12530D | ANS/TT18 | AT4G22880 | 2.3 | 0.E+00 | proanthocyanin biosynthesis |
| rs1114 | A01 | BnaA01g25630D | DELTAVPE | AT3G20210 | 159.134 | 4.00E-54 | seed coat development |
| rs1703 | A01 | BnaA01g31810D | SEC8 | AT3G10380 | 181.31 | e-106 | mucilage biosynthetic process involved in seed coat development |
| rs4459 | A02 | BnaA02g05900D | CSLA2 | AT5G22740 | 4.8 | e-131 | mucilage metabolic process involved in seed coat development |
| rs4806 | A02 | BnaA02g10250D |  | AT5G53500 | 1.744 | E-120 | signal transduction |
| rs3461 | A02 | BnaA02g26180D |  | AT2G02060 | 5.678 |  | regulation of transcription |
| rs4157 | A02 | BnaA02g34360D | CESA9 | AT2G21770 | 213.57 | 1.00E-62 | cell wall organization, cellulose biosynthetic process, plant-type cell wall biogenesis, seed coat development |
| rs4156 | A02 | BnaA02g34360D | CESA5 | AT5G09870 | 209.44 | 0.E+00 | cellulose biosynthetic process, cellulose metabolic process,anthocyanin accumulation |
| rs7485 | A03 | BnaA03g08640D | CSLA2 | AT5G22740 | 41 | e-132 | mucilage metabolic process involved in seed coat development |
| rs7538 | A03 | BnaA03g09510D | ACT4 | AT5G59370 | 29.28 | 0.E+00 | actin cytoskeleton organization, cytoskeleton organization, gluconeogenesis |
| rs8158 | A03 | BnaA03g17130D | TTG2 | AT2G37260 | 315.85 | e-129 | seed coat development |
| rs5756 | A03 | BnaA03g29980D | CHIP | AT3G07370 | 160.45 | 2.00E-44 | signal transduction |
| rs6213 | A03 | BnaA03g39500D | TT16 | AT5G23260 | 211.81 | 2.00E-37 | anthocyanin accumulation in tissues in response to UV light, carpel development, flavonoid biosynthetic process |
| rs7161 | A03 | BnaA03g52020D | CESA10 | AT2G25540 | 86.25 | 1.00E-92 | cell wall organization, cellulose biosynthetic process |
| rs7161 | A03 | BnaA03g52020D | CESA1 | AT4G32410 | 86.25 | 0.E+00 | cell wall organization, cellulose biosynthetic process |
| rs8652 | A04 | BnaA04g16590D |  | AT2G28660 | whithin | 1.00E-46 | positive regulation of flavonoid biosynthetic process |
| rs12067 | A05 | BnaA05g05940D | EXO70H2 | AT2G39380 | 3.028 | 0.E+00 | amino acid transport |
| rs12234 | A05 | BnaA05g07950D |  | AT2G36350 | whithin | 0.E+00 | determination of meristem maintenance |
| rs24229 | A05 | BnaA05g08650D | PLL1 | AT2G35350 | whithin | 4.00E-97 | protein palmitoylation，proteolysis |
| rs12423 | A05 | BnaA05g10540D | RLP27 | AT2G33060 | whithin | 0.E+00 | defense response, signal transduction |
| rs10412 | A05 | BnaA05g14410D | MUM4 | AT1G53500 | 23.87 | 0.E+00 | seed coat development |
| rs11205 | A05 | BnaA05g20600D | CAD4 | AT3G19450 | 93.06 | e-140 | lignin biosynthetic process |
| rs11344 | A05 | BnaA05g23110D | PLDP1 | AT3G16785 | whithin | 0.E+00 | signaling pathway |
| rs11682 | A05 | BnaA05g27640D | GPAT5 | AT3G11430 | 252.54 | 0.E+00 | suberin biosynthetic process |
| rs11683 | A05 | BnaA05g27640D | GPAT5 | AT3G11430 | 253.31 | 0.E+00 | suberin biosynthetic process |
| rs11682 | A05 | BnaA05g28060D | CESA3 | AT5G05170 | 165.89 | 3.00E-93 | anthocyanin accumulation, cell wall biogenesis, cell wall organization, cellulose biosynthetic process |
| rs11683 | A05 | BnaA05g28060D | CESA3 | AT5G05170 | 165.95 | 3.00E-93 | anthocyanin accumulation, cell wall biogenesis, cell wall organization, cellulose biosynthetic process |
| rs11682 | A05 | BnaA05g28180D | SUN2 | AT3G10730 | 128.93 | e-145 | protein homooligomerization |
| rs11683 | A05 | BnaA05g28180D | SUN2 | AT3G10730 | 128.98 | e-145 | protein homooligomerization |
| rs11682 | A05 | BnaA05g28440D | SEC8 | AT3G10380 | 20.49 | e-111 | mucilage biosynthetic process involved in seed coat development |
| rs11683 | A05 | BnaA05g28440D | SEC8 | AT3G10380 | 20.54 | e-111 | mucilage biosynthetic process involved in seed coat development |
| rs11682 | A05 | BnaA05g28470D | PAL4 | AT3G10340 | 9.17 | 0.E+00 | L-phenylalanine catabolic process, cinnamic acid biosynthetic process |
| rs11683 | A05 | BnaA05g28470D | PAL4 | AT3G10340 | 9.22 | 0.E+00 | L-phenylalanine catabolic process, cinnamic acid biosynthetic process |
| rs11682 | A05 | BnaA05g28470D | PAL3 | AT5G04230 | 9.17 | 9.00E-56 | L-phenylalanine catabolic process, cinnamic acid biosynthetic process |
| rs11683 | A05 | BnaA05g28470D | PAL3 | AT5G04230 | 9.22 | 9.00E-56 | L-phenylalanine catabolic process, cinnamic acid biosynthetic process |
| rs11690 | A05 | BnaA05g28570D |  | AT3G10200 | whithin | 6.00E-87 | integral component of membrane |
| rs11700 | A05 | BnaA05g28760D | CCR1 | AT3G09780 | 9.04 | 0.E+00 | fatty acid catabolic process |
| rs14751 | A06 | BnaA06g11740D | TRFL3 | AT1G17460 | whithin | 8.00E-65 | seed dormancy process |
| rs14859 | A06 | BnaA06g13770D | GAMMA CA1 | AT1G19580 | whithin | 8.00E-34 | starch biosynthetic process |
| rs12881 | A06 | BnaA06g20980D |  | AT3G49320 | whithin | 3.00E-67 | unknown |
| rs19419 | A07 | BnaA07g03960 |  | AT5G02860 | whithin | 0.E+00 | unknown |
| rs17083 | A07 | BnaA07g07350D |  | AT1G30370 | 348.89 | 0.E+00 | seed coat development |
| rs15053 | A07 | BnaA07g12690D | VND3 | AT5G66300 | 2.1 | 5.00E-77 | regulation of transcription |
| rs4506 | A07 | BnaA07g15220D |  | AT5G41040 | 16.81 | 0.E+00 | cell wall pectin biosynthetic process, suberin biosynthetic process |
| rs15575 | A07 | BnaA07g19780D |  | AT1G80000 | whithin | 7.00E-91 | unknown |
| rs26534 | A07 | BnaA07g20380D | MC7 | AT1G79310 | whithin | 7.00E-51 | proteolysis |
| rs15766 | A07 | BnaA07g21800D |  | AT1G75210 | whithin | 2.00E-73 | unknown |
| rs16541 | A07 | BnaA07g34460D | CPuORF56 | AT1G78882 | whithin | 2.00E-73 | unknown |
| rs17631 | A08 | BnaA08g01300D | ERF8 | AT1G53170 | 6.80 | 1.00E-54 | signaling pathway |
| rs50399 | A08 | BnaA08g07370D |  | AT5G32470 |  | e-119 | unknown |
| rs31887 | A08 | BnaA08g15930D | CAD-B2 | AT4G37990 | 298.39 | e-175 | lignin biosynthetic process |
| rs18184 | A08 | BnaA08g18900D |  | AT1G27530 | whithin | 3.00E-90 | unknown |
| rs18388 | A08 | BnaA08g23220D | AHA10 | AT1G17260 | 102.24 | 7.00E-91 | proanthocyanidin biosynthetic process |
| rs18390 | A08 | BnaA08g23390D | CYP72C1 | AT1G17060 | 142.26 | e-136 | brassinosteroid biosynthetic process |
| rs19856 | A09 | BnaA09g04450D | GATA12 | AT5G25830 | 0.716 | 5.00E-61 | positive regulation of transcription |
| rs21777 | A09 | BnaA09g11620D |  | AT1G64370 | 9.225 | 2.00E-71 | unknown protein |
| rs19431 | A09 | BnaA09g15710D | TT3 | AT5G42800 | 75.18 | 3.00E-69 | anthocyanin-containing compound biosynthetic process |
| rs20236 | A09 | BnaA09g25550D | VSR6 | AT1G30900 | whithin | 0.E+00 | response to fructose, response to glucose |
| rs17505 | A09 | BnaA09g26150D |  | AT1G30370 | 297.29 | 0.E+00 | seed coat development |
| rs20500 | A09 | BnaA09g31780D | F3H TT6 | AT3G51240 | 41.49 | e-160 | anthocyanin-containing compound biosynthetic process, flavonoid biosynthetic process |
| rs20589 | A09 | BnaA09g31780D | F3H TT6 | AT3G51240 | 283.7 | e-160 | anthocyanin-containing compound biosynthetic process, flavonoid biosynthetic process |
| rs20718 | A09 | BnaA09g35490D | CCR3 | AT3G55950 | 213.88 | 0.E+00 | egulation of plant-type hypersensitive respons |
| rs21223 | A09 | BnaA09g45190D | CYP72C1 | AT1G17060 | 186.47 | e-140 | brassinosteroid biosynthetic process |
| rs8570 | A10 | BnaA10g05890D |  | AT5G51040 | whithin | 3.00E-38 | unknown protein |
| rs51207 | A10 | BnaA10g09020D | TT15 | AT1G43620 | 157.87 | 1.00E-80 | flavonoid biosynthetic process, seed coat development |
| rs23703 | A10 | BnaA10g11420D | AHA3 | AT5G57350 | 30.95 | 0.E+00 | glucosinolate biosynthetic process |
| rs36486 | A10 | BnaA10g23330D | TT7 | AT5G07990 | 319.58 | e-166 | anthocyanin-containing compound biosynthetic process, flavonoid biosynthetic process |
| rs39096 | C01 | BnaC01g11820D |  | AT4G19460 | 94.46 | 0.E+00 | galactolipid biosynthetic process |
| rs39096 | C01 | BnaC01g11870D |  | AT4G19500 | 47.72 | 0.E+00 | signal transduction |
| rs39096 | C01 | BnaC01g11870D |  | AT4G19510 | 47.72 | 0.E+00 | signal transduction |
| rs37192 | C01 | BnaC01g17160D | RHS15 | AT4G25220 |  | 0.E+00 | transmembrane transport |
| rs35374 | C01 | BnaC01g18230D |  | AT4G26180 | whithin | e-131 | Encodes a mitochondrial CoA transporter |
| rs30941 | C02 | BnaC02g25560D |  | AT1G79190 | 9.96 | e-159 | unknown |
| rs41410 | C02 | BnaC02g28050D | GATL5 | AT1G02720 | 487.01 | e-106 | seed coat development |
| rs41410 | C02 | BnaC02g28050D | GATL6 | AT4G02130 | 487.01 | 0 | seed coat development |
| rs41439 | C02 | BnaC02g28390D |  | AT4G12290 | whithin | 0.E+00 | amine metabolic process, oxidation-reduction process |
| rs31106 | C02 | BnaC02g28920D |  | AT4G11030 | 1.678 | 2.00E-66 | fatty acid biosynthetic process |
| rs33699 | C02 | BnaC02g30750D | CESA4 | AT5G44030 | 2.04 | 9.00E-33 | cell wall biogenesis, cell wall macromolecule metabolic process, cellulose biosynthetic process |
| rs7948 | C03 | BnaC03g18320D | ZFN2 | AT2G32930 | 0 | 7.00E-37 | regulation of transcription |
| rs27904 | C03 | BnaC03g23150D | CRK1 | AT2G41140 | whithin | 8.00E-96 | protein autophosphorylation，proteolysis |
| rs50423 | C03 | BnaC03g54750D | FAR5 | AT3G44550 | 6.88 | 2.00E-90 | suberin biosynthetic process |
| rs29836 | C03 | BnaC03g62730D |  | AT4G18570 | whithin | e-116 | proteolysis |
| rs39277 | C04 | BnaC04g11650D | RLP26 | AT2G33050 | 0.16 | 0.E+00 | defense response, signal transduction |
| rs42140 | C04 | BnaC04g16150D |  | AT2G28130 | whithin | 4.00E-51 | unknown protein |
| rs42161 | C04 | BnaC04g16320D |  | AT1G42470 | whithin | 7.00E-80 | hedgehog receptor activity |
| rs42895 | C04 | BnaC04g26110D |  | AT3G54690 | 1.415 | 7.00E-70 | carbohydrate metabolic process |
| rs8403 | C04 | BnaC04g36500D |  | AT2G24360 | whithin | 0.E+00 | protein phosphorylation |
| rs33387 | C04 | BnaC04g41700D |  | AT2G31010 | whithin | e-119 | protein phosphorylation，proteolysis |
| rs46100 | C04 | BnaC04g43280D | LUH | AT2G32700 | whithin | 2.00E-84 | mucilage pectin biosynthetic process |
| rs8970 | C04 | BnaC04g44680D | CSLA7 | AT2G35650 | whithin | e-162 | signaling pathway |
| rs45726 | C05 | BnaC05g07940D |  | AT1G10417 | whithin | 7.00E-41 | unknown |
| rs32622 | C05 | BnaC05g23260D |  | AT1G30370 | 250.08 | 0.E+00 | seed coat development |
| rs35157 | C05 | BnaC05g36050D |  | AT3G17410 | whithin | e-100 | galactolipid biosynthetic process |
| rs38938 | C05 | BnaC05g37890D |  | AT5G22250 | 96.67 | e-174 | intracellular signal transduction |
| rs46514 | C05 | BnaC05g39780D |  | AT3G13540 | 283.911 | 1.00E-61 | seed coat development |
| rs37369 | C05 | BnaC05g42720D | SEC8 | AT3G10380 | 102.05 | e-114 | mucilage biosynthetic process involved in seed coat development |
| rs37369 | C05 | BnaC05g42720D | SUN2 | AT3G10730 | 72.41 | e-109 | protein homooligomerization |
| rs37338 | C05 | BnaC05g43230D | CCR1 | AT3G09780 | 18.57 | 0.E+00 | fatty acid catabolic process |
| rs47605 | C07 | BnaC07g05320D |  | AT2G15900 | 7.251 | e-120 | signal transduction |
| rs28562 | C07 | BnaC07g37950D | TT18/ANS | AT4G22880 | 32.06 | 0.00E+00 | oxidation-reduction process, proanthocyanidin biosynthetic process |
| rs28562 | C07 | BnaC07g37950D | CAD2 | AT4G23100 | 167.96 | 1.00E-54 | cellulose biosynthetic process |
| rs29256 | C07 | BnaC07g47260D | CAD2 | AT2G21730 | 102.7 | 1.00E-28 | lignin biosynthetic process, oxidation-reduction process |
| rs29256 | C07 | BnaC07g47260D | CAD9 | AT4G39330 | 102.7 | e-169 | lignin biosynthetic process, oxidation-reduction process |
| rs29256 | C07 | BnaC07g47260D | CAD3 | AT4G39330 | 102.7 | 8.00E-33 | lignin biosynthetic process, oxidation-reduction process |
| rs48993 | C08 | BnaC08g05590D | scpl44 | AT1G43780 | 35.175 | 5.00E-79 | proteolysis |
| rs41926 | C08 | BnaC08g11060D | RPB1 | AT4G35800 | whithin | 0.E+00 | gene silencing by RNA, hydrogen peroxide biosynthetic process |
| rs18448 | A08 | BnaC08g11760D |  | AT4G24690 | whithin | 1.00E-70 | protein polymerization，proteolysis |
| rs18404 | C08 | BnaC08g17160D | CYP72C1 | AT1G17060 | 7.878 | e-121 | brassinosteroid biosynthetic process |
| rs30489 | C08 | BnaC08g17270D | RLP3 | AT1G17250 | 2.51 | 0.E+00 | signal transduction |
| rs30489 | C08 | BnaC08g17280D | AHA10 | AT1G17260 | 0.426 | 7.00E-91 | proanthocyanidin biosynthetic process |
| rs31652 | C08 | BnaC08g44970D | CCR2 | AT1G06820 | 73.71 | 6.00E-57 | carotenoid biosynthetic process |
| rs31693 | C08 | BnaC08g46020D | GATL5 | AT1G02720 | 207.57 | e-105 | seed coat development |
| rs32540 | C09 | BnaC09g01820D | CYP81D1 | AT3G28740 | 2.347 | e-146 | oxidation-reduction process |
| rs39756 | C09 | BnaC09g17150D | TT3 | AT5G42800 | 210.99 | 2.00E-64 | anthocyanin-containing compound biosynthetic process |
| rs39358 | C09 | BnaC09g18860D | CYP707A3 | AT5G45340 | whithin | e-130 | abscisic acid catabolic process, oxidation-reduction process |
| rs38997 | C09 | BnaC09g36340D | CSLA2 | AT5G22740 | 116.81 | e-126 | mucilage metabolic process involved in seed coat development |
| rs38997 | C09 | BnaC09g36490D | FAR1 | AT5G22500 | 286.74 | 1.00E-48 | suberin biosynthetic process |
| rs32426 | C09 | BnaC09g40740D | TT19 | AT5G17220 | 289.54 | e-118 | anthocyanin-containing compound biosynthetic process, regulation of flavonol biosynthetic process |
| rs47303 | C09 | BnaC09g43250D | CHS/TT4 | AT5G13930 | 15.19 | 0.E+00 | flavonoid biosynthetic process |
